# 5.4 BIOLOGICAL AND EPIDEMIOLOGICAL EXAMINATION OF TRANSDIAGNOSTIC AND SPECIFIC SYMPTOM DIMENSIONS AT PSYCHOSIS ONSET: FINDINGS FROM THE EUGEI STUDY

**DOI:** 10.1093/schbul/sby014.016

**Published:** 2018-04-01

**Authors:** Diego Quattrone, Pak Sham, Evangelos Vassos, Charlotte Gayer-Anderson, Giada Tripoli, Laura Ferraro, Michael O’Donovan, Alex Richards, Jim van Os, Craig Morgan, Ulrich Reininghaus, Robin Murray, Marta Di Forti, Cathryn Lewis

**Affiliations:** 1 Institute of Psychiatry, Psychology & Neuroscience, King’s College London; 2 Centre for Genomic Sciences, The University of Hong Kong; 3 University of Palermo; 4 MRC Centre for Neuropsychiatric Genetics and Genomics, Cardiff University; 5 School of Mental Health and Neuroscience, Maastricht University

## Abstract

**Background:**

Current diagnostic models of psychosis have been questioned since Kraepelin’s original dichotomy of dementia praecox and manic depression. Indeed, increasing evidence has suggested that a dimensional approach might be a valid alternative platform for research. However, while an increasing number of studies have investigated how environmental risk factors for affective and non-affective psychosis map onto symptom dimensions, only a few have examined these dimensions in relation to genetic variants as summarised by Polygenic Risk Score (PRS). Furthermore, no studies have examined the putative effect of PRS for Schizophrenia (SZ), Bipolar Disorder (BP), and Major Depressive Disorder (MDD) on previously identified general and specific symptom dimensions. At the same time, only one study has investigated how symptoms vary according to epidemiological factors such as living in urban neighbourhoods. The objectives of this study were to: 1) test whether a bi-factor model statistically fits the conceptualization of psychosis as composed of general and specific dimensions; 2) examine the extent to which SZ, BP, and MDD PRSs explain the phenotypic variance due to general and specific dimensions; 3) test the hypothesis that the general psychosis dimension would be more severe in highly urban environments.

**Methods:**

We used clinical and epidemiological data from the EUropean network of national schizophrenia networks studying Gene-Environment Interactions (EUGEI) study, including 2322 First Episode Psychosis (FEP) patients recruited in 17 sites across 6 countries. Genetic variants were collectively analyzed for 800 individuals.

The following analysis steps were performed:

1) Psychopathology items were analysed using multidimensional item response modelling in MPlus to estimate unidimensional, multidimensional, and bi-factor models of psychosis. Model fit statistics included Log-Likelihood, and Akaike and Bayesian Information Criteria to compare these models.

2) SZ, BP, and MDD PRSs for general and specific dimensions were built using PRSice. Summary statistics from large case-control mega-analyses from the Psychiatric Genomics Consortium were used as base data sets and general and specific dimension scores were used as discovery data sets. Individuals’ number of risk alleles in the discovery sample was weighted by the log odds ratio from the base samples, accounting for population stratification, and summed into the three PRSs.

3) Multilevel regression analysis was used in STATA 14 to examine the variance in general dimension due to the population density levels across the sites.

**Results:**

A bi-factor solution, composed of one general and five specific symptom dimensions, showed the best model fit statistics.

Higher SZ PRS score was associated with higher scores on positive dimensions (β= 0.27, t=2.11, p<0.05); higher BP PRS was associated with higher scores on mania dimension (β= 0.17, t=2.11, p<0.05); higher MDD PRS was associated with lower scores on negative dimension (β= -0.31, t=-2.25, p<0.05). No trends of association were found for SZ, BP, or MDD PRSs and the general psychosis dimension.

The transdiagnostic symptom dimension score was elevated in people living in more densely populated sites (η2=0.077, 95% CI 0.057–0.098).

**Discussion:**

Our results suggest that a) symptom dimension structure at FEP is best represented by the bi-factor model; b) in FEP patients, there is a trend of associations between SZ PRS and positive dimension, and between BP PRS and mania dimension; and c) elevated level of transdiagnostic symptomatology was observed in more densely populated sites.

